# Challenges and Solutions to Pre- and Post-Randomization Subgroup Analyses

**DOI:** 10.1007/s11886-014-0531-2

**Published:** 2014-08-19

**Authors:** Manisha Desai, Karen S. Pieper, Ken Mahaffey

**Affiliations:** 1 Quantitative Sciences Unit, Department of Medicine, Stanford University, 1070 Arastradero Road #305, Palo Alto, CA 94306 USA; 2 Duke Clinical Research Institute, PO Box 17969, Durham, NC 27715 USA; 3 Department of Medicine, Stanford University, 300 Pasteur Drive, Stanford, CA 94305 USA

**Keywords:** Subgroup analyses, Post-randomization, Causal inference, Multiplicity, Tests of interaction, Bias, A priori hypotheses

## Abstract

Subgroup analyses are commonly performed in the clinical trial setting with the purpose of illustrating that the treatment effect was consistent across different patient characteristics or identifying characteristics that should be targeted for treatment. There are statistical issues involved in performing subgroup analyses, however. These have been given considerable attention in the literature for analyses where subgroups are defined by a pre-randomization feature. Although subgroup analyses are often performed with subgroups defined by a post-randomization feature—including analyses that estimate the treatment effect among compliers—discussion of these analyses has been neglected in the clinical literature. Such analyses pose a high risk of presenting biased descriptions of treatment effects. We summarize the challenges of doing all types of subgroup analyses described in the literature. In particular, we emphasize issues with post-randomization subgroup analyses. Finally, we provide guidelines on how to proceed across the spectrum of subgroup analyses.

## Introduction

Subgroup analyses are those that aim to estimate treatment effects within subgroups of patients enrolled in a clinical trial. They are performed frequently [1]. For example, Pocock and others found in their review of comparative clinical trials that 51 % of the trials described in three high-impact journals performed at least one subgroup analysis [2]. In a more recent review, Parker and others found that 58 of 67 clinical trials evaluating cardiovascular events performed subgroup analyses [3]. Reasons that motivate such analyses include illustrating that the treatment effect is consistent across different patient characteristics or identifying those patient characteristics that should be targeted for treatment (e.g., gender, if females respond well to the experimental therapy, whereas males do not). Many publications of randomized clinical trials include a forest plot, which displays the treatment effect across a series of subgroups [4].

In their seminal paper on the topic, Yusuf and others categorize subgroup analyses as those that are “proper” and those that are “improper” [5]. The former involves subgroups defined by a baseline characteristic that cannot be influenced by treatment, whereas the latter defines subgroups on a post-randomization feature, which may potentially be influenced by the treatment itself. An analysis that examines effects by gender, for example, falls into the category of proper subgroup analyses. A common example of an improper subgroup analysis is a “per-protocol” analysis, defined as an analysis comparing outcomes by treatment group among those who adhered to the assigned protocol—a feature measured after initiation of the study. This is performed with the goal of drawing inference on the potential biological effect of the experimental treatment. Because adherence (or any post-randomization feature) may be influenced by the actual treatment assigned, however, this can lead to potentially biased estimates of treatment risk or benefit.

Much attention has been paid to proper or pre-randomization subgroup analyses (e.g., [5–8]). In particular, statisticians have raised concerns about the risk of misinterpreting findings resulting from such analyses [2]. Clinical investigators have in turn expressed unease that by maintaining statistical rigor, important scientific discoveries could be missed (e.g., [9, 10]). We argue, however, that to make good scientific discoveries, one must apply valid statistical principles. To respond to the controversy, Yusuf and others developed guidelines to consider when doing such analyses [5]. Importantly, the guidelines only applied to proper subgroup analyses. The authors advised against performing post-randomization subgroup analyses, as the potential for presenting biased treatment estimates is high. Similarly, Assmann and others provided guidelines that applied to the proper setting only, echoing the concerns raised by Yusuf et al. and advising against presenting findings from post-randomization subgroup analyses [5, 6]. In fact, because these analyses are deemed problematic, many investigators reviewing the state of subgroup analyses neglected discussion of post-randomization subgroup analyses in their reports (e.g., [2, 6, 7]). Hirji et al. noted, however, that not addressing issues specific to post-randomization subgroup analyses has not deterred their use or, perhaps more importantly, their influence on clinical practice [1]. We generally agree that the issues faced by both types of analyses should be addressed; investigators continue to perform post-randomization subgroup analyses and clinicians are faced with the task of interpreting the findings, which may ultimately have influence on clinical practice.

The goal of our paper is to summarize the challenges and pitfalls in doing subgroup analyses described in the literature. To that end, we summarize and expand upon guidelines and solutions presented by previous investigators. Our paper emphasizes issues with post-randomization subgroup analyses as these pose the additional complexity of handling potential bias. Unfortunately, issues specific to these analyses have also been neglected by the clinical literature, as pointed out by Hirji and others [1]. There are currently no guidelines for how to perform and report on post-randomization subgroup analyses. Ours is the first to provide guidelines on how to proceed across the spectrum of subgroup analyses.

To that end, we searched for papers on PubMed using the term “subgroup analysis” and chose those 21 papers spanning the years 1987 to 2013 and tabulated challenges posed, guidelines presented, and solutions provided [1–3, 5–21, 22•].

## Challenges Encountered in Subgroup Analyses

Common pitfalls and challenges identified in previous studies include increased type I error rates resulting from testing multiple hypotheses, increased type II error rates caused by testing hypotheses for which the study was not designed, incorrect application of statistical tools for assessing heterogeneity across subgroups, testing data-driven (as opposed to pre-specified) hypotheses, performing subgroup analyses when overall findings are negative, considering hypotheses not motivated by biology, and potential bias from performing subgroup analyses where subgroups are defined by a post-randomization feature. All of these challenges adversely impact interpretation. Table 1 summarizes the corresponding statistical implications of these challenges with high-level solutions, which we describe in greater detail below.

**Table 1:** Recognized pitfalls and solutions for subgroup analysis as described in the literature

Statistical issue	Potential solutions
Increased type I error rate (false-positive findings)	Limit the number of analyses performed to those that are pre-specified and biologically plausible Adjust for multiplicity
Increased type II error rate (low power to detect interesting findings)	Design study to examine particularly relevant subgroups
Biased estimates of treatment	Limit to performing proper analyses
Findings are difficult to interpret	Emphasize overall findings Utilize formal tests of interaction to appropriately assess heterogeneity of effects Do not perform subgroup analyses if overall findings are negative Report the number of tests performed Report the number of tests that were specified a priori and the number that were specified a posteriori Report all findings—positive and negative

Many of the issues raised are interrelated. Knowing whether an analysis was pre-specified (or *specified a priori*) versus data-driven (or a posteriori) is important to consider for appropriate interpretation. A posteriori tests are circular in that one can examine differences in effect by many possible subgroups, find something interesting, and then formulate a hypothesis to test the interesting finding. In this way, the testing leads to a hypothesis of interest to be tested. If the purpose of the examination of the data is to generate hypotheses, the newly generated hypothesis should be tested on an independent data set and not the data set upon which the hypothesis was generated. A priori tests, in contrast, are pre-specified—typically based on biological understandings and/or previous findings in the literature. The hypothesis has been formulated prior to examination of the data, and therefore, the results support or refute the hypothesis rather than the other way around. Thus, knowledge of which type of analysis was performed provides context in which to interpret the findings [5, 11, 12, 17].

Along similar lines, several authors discuss the importance of *biological plausibility* as one of the criterion for performing subgroup analyses [11, 17]. Altman [16], however, believes this is the weakest of the four criteria put forward by Furberg and Byington [23] to providing credibility for a finding, as he believes clinical investigators can always find a biologically plausible explanation for any finding.

The issues described above (a priori specification and biological plausibility) are both related to another issue—that of *multiplicity* or *multiple testing*. Several authors raised this issue, which affects interpretation in that it can lead to false-positive findings and/or an overemphasis of findings [1, 5, 7, 16, 20]. Key to appropriate interpretation is knowledge of how many subgroup analyses have been performed. Note that even when there is no difference in treatment effect by subgroups, when testing 20 uncorrelated hypotheses at the 0.05 level of significance, we would expect 1 of these tests to be incorrectly rejected (or falsely positive). Compare an evaluation of 2 hypotheses where one was found positive, to an evaluation of 20 hypotheses where 1 was found positive. We can have more confidence in the positive test that was based on 2 hypotheses than the positive test from 20. Statistically, we can more formally address this by using methods to appropriately control the family-wise error rate (e.g., through use of a Bonferroni correction) or the false-discovery rate depending on the research question [24, 25].

Prevalent in the literature is a tendency to incorrectly assess heterogeneity of treatment effects [1, 2, 5–7, 16, 21]. This is done by testing for treatment effects within each level of the subgroup of interest (e.g., evaluating the treatment effect among males and among females if gender is of interest) and then comparing *p* values or point estimates across subgroups informally to determine whether effects are heterogeneous. This is problematic, as some random heterogeneity is expected across factors. Whether the observed heterogeneity is larger than expected by chance needs to be addressed through *formal use of statistical tests of interaction* like the Breslow-Day test, the Mantel-Haenszel test, or the interaction test from a modeling procedure [26].

Clinical trials are rarely designed to evaluate differences in treatment effect in subgroups, yielding *low power* to investigate such differences and increasing the type II error rate of the hypothesis. In other words, if the study is not designed to discern differences in heterogeneity across subgroups, there is a high probability of incorrectly concluding there are no differences even when there are [5]. For example, suppose a 2-arm study investigating a new experimental therapy for breast cancer requires 240 patients or 120 subjects per arm to have 80 % power to detect a hazard ratio of 1.5 assuming 50 % of the subjects in the standard treatment arm are event free at 6 months. If we wanted to examine whether these differences varied by low- versus high-grade tumors and if we assumed that half of enrolled patients would be diagnosed with low-grade tumors, we would need 449 patients per arm to have 80 % power to detect differential treatment effects by grade assuming no difference in hazard rates between treatment arms among those diagnosed with low-grade tumors and a hazard ratio of 2 for those diagnosed with high-grade tumors. With a sample size of 240, there is less then half the chance (48 %) to detect differential treatment effects by grade. Further, if the proportion of subjects with high- versus low-grade tumors is imbalanced, as is typically the case, this will require an even larger increase in the sample size. Suppose 70 % of those enrolled have high-grade tumors and only 30 % have low-grade tumors, then 517 subjects would be required to have 80 % power to detect this difference in hazard ratios, where the current design only gives a 40 % chance of detecting the difference. Thus, unless there is strong a priori evidence to motivate redesigning the trial, the study will not be designed to assess differences across most subgroups. Not having sufficient evidence to support heterogeneity of effects with such a high type II error rate could mislead investigators into concluding effects are homogenous.

Concerns have been raised in *performing subgroup analyses when the overall trial results did not achieve statistical significance for the primary hypothesis* [2, 7, 11]. Often, these analyses are performed to salvage a “negative” trial or to learn from the trial about future areas of investigation. This may create confusion for the reader and is generally not recommended. Bulpitt specified four criteria that should hold before subgroup analyses are pursued, which included that the overall findings be positive before proceeding, where the other three were that the hypotheses were biologically plausible, pre-specified, and that the analysis was not inherently subject to bias [11]. If the overall findings are negative, the question of which subgroups benefit more or less is no longer relevant and any so-called positive findings based on subgroup analyses are then challenging to interpret.

Another issue that impedes interpretation is *selective reporting*. Whereas not all findings warrant detailed reporting, all analyses performed do. In order for the reader to have an appropriate context to interpret the findings presented by the author as interesting or relevant, the analyses that were performed, the number, whether they were pre-specified, and which were negative and positive should be provided [2].

Specific to post-randomization subgroup analyses is the issue of *bias*, requiring special attention to be paid to the methods employed and interpreted. Pre-randomization subgroup analyses compare subjects by baseline features which are unaffected by treatment assignment. Consequently, in theory, these subgroups should be comparable because randomization principles should still apply. It is standard for most clinical trials to specify an intent-to-treat (ITT) analysis as the primary analysis. Such an analysis can be described as an inclusive analysis. All subjects randomized to treatment are included in the analysis and analyzed by their randomized treatment assignment regardless of adherence. Because it maintains randomization, it has good statistical properties for testing the null hypothesis of no treatment effect [27]. Many investigators are uncomfortable accepting these ITT findings without further analyses, however, because of issues related to adherence or loss-to-follow-up. A post-randomization subgroup analysis called a “per-protocol” analysis is consequently proposed, where only those subjects who fulfill the protocol in terms of eligibility, interventions, and assessments are included in the analysis. Those who complete the trial and adhere to the assigned protocol, however, are typically different from those who do not, and this can induce a bias when estimating the treatment effect. For example, suppose a study is evaluating the effect of a new experimental therapy (arm A) on reducing cardiovascular events relative to standard therapy, and suppose that arm A is actually harmful. As a result, those assigned to arm A are more likely to observe changes in their lipid profile and, consequently, stop adhering to the study drug, making them more likely to be excluded from the analysis. This may make arm A look more favorable or less harmful than it actually is.

Pieper and others illustrated the danger of post-randomization subgroup analyses with another important example in cardiovascular medicine [20]. While many believe that treatment of platelet glycoprotein (GP) IIb/IIIa inhibition should be used only in acute coronary syndrome (ACS) patients undergoing percutaneous coronary intervention (PCI), Pieper and others argue that the evidence to support this is based on problematic subgroup analyses that fall into the improper category and that therefore yield potentially misleading descriptions of treatment effects. In clinical trials evaluating the benefit of GP in non-ST-elevation ACS patients, questions of which subtypes of patients would most benefit have been addressed through series of subgroup analyses. One targeted subgroup has been those patients undergoing PCI versus those receiving medical management only. Specifically, whether the benefits differ between these two groups of ACS patients has been a subject of debate. Because undergoing PCI is not a characteristic known at baseline, but rather defined by an event that occurs at some point post-randomization, subjects in subgroups defined by the performance of a PCI or not post-randomization may differ by factors other than PCI. For example, this subgroup assignment may relate to their underlying disease and/or importantly may have been influenced by the treatment itself. Suppose GP inhibitors make PCI less necessary so that those in the control group undergo PCI at a higher rate, and further that those undergoing PCI in the treatment group are inherently different from those undergoing PCI in the control group. In general, if the reason for PCI differs for subjects exposed to GP inhibitors versus those in the control group, this can make PCI patients exposed to treatment fundamentally different from those not exposed to treatment, potentially confounding the treatment effect.

Below, we discuss some possible solutions to minimize or address bias inherent in post-randomization subgroup analyses.

## Potential Solutions for Addressing or Minimizing Bias Encountered in Post-Randomization Subgroup Analyses

There are statistical tools that can be used to minimize potential bias induced by post-randomization subgroup analyses. For evaluating whether GP inhibitors reduce ischemic events in patients with ACS by PCI versus medical management, Pieper and others offer four alternative solutions to the standard analytic approach of post-randomization subgroups [20]. In a standard subgroup analysis, the assignment of a patient to the PCI subgroup is treated as static (i.e., if a patient undergoes PCI at any point during the observation period, the patient is placed in the PCI subgroup. Otherwise, the patient is considered to have undergone medical management only). This categorization can be problematic particularly if an event such as a myocardial infarction (MI) occurred prior to receiving PCI (i.e., during the period when the patient had only received medical management), and furthermore, the MI may have influenced the patient to have the PCI. To address this issue, the authors suggest the following four approaches:

- *Classify subjects as in the standard approach but exclude any events that occurred prior to the PCI*. By doing this, the authors minimize bias due to potential confounding by indication.
- *Analyze subjects during the medical management period only*, *so that subjects are observed until the first occurrence of the relevant ischemic event*, *PCI*, *or the end of study*, *whichever occurs first*, *using survival analytic techniques.* In this solution, the treatment effect is evaluated under medical management. One cannot, however, evaluate the differential treatment effect for those who undergo PCI versus medical management. Like the first solution, however, this approach offers a method to evaluate the treatment effect by minimizing potential confounding of PCI.
- *Consider PCI as a time*-*varying variable and make use of survival analytic techniques that incorporate time*-*dependent confounders.* In such an analysis, the subject contributes data to the medical management group during the time period prior to the subject’s PCI. If the subject had a PCI, the subject contributes data to the PCI group after this point. The authors point out that this approach does not address inherent selection bias, however.
- *Directly address the selection bias through use of propensity score methods* [28]. Under certain assumptions, such an approach would also allow evaluation of heterogeneity of effects between PCI and medical management groups through use of an interaction term.

The challenge of bias faced by post-randomization subgroup analyses are similar to those that many observational studies face, particularly those in the longitudinal setting, and as Pieper and others suggest, we can borrow tools used in those settings for drawing causal inference while being mindful of their limitations [20]. The idea behind the proposed propensity score method is that one can use observed information to model the propensity for the exposure or treatment of interest and use this to adjust for confounding between groups of interest. There are numerous ways in which propensity scores can be incorporated into the analysis (e.g., through matching in the design, stratification, through inclusion as a covariate in the model, or inverse probability weighting) [29•]. An interaction term between PCI and treatment can then evaluate whether the treatment effect differs for those who do and do not undergo PCI. Validity of the results relies on an assumption that the propensity for undergoing PCI can be modeled by observed variables only. Under this assumption, the estimated treatment effect is unbiased. However, this particular approach only includes baseline characteristics to predict PCI use. Newer methods have been developed and will be discussed, which allow the propensity to change over time.

Other causal inference tools that can be applied include the use of instrumental variables or, more generally, principal stratification methods, and marginal structural models [14, 15, 30, 31]. The idea behind instrumental variables is to identify an instrument or variable that has a causal relationship with treatment assignment but no direct causal relationship to the outcome to use in evaluating the effect of interest. This approach is discussed in detail in the context of assessing treatment effects among compliers and relies upon strong assumptions involving the instrument [14]. Marginal structural models can be used in this context as well. The idea behind marginal structural models is to weigh subjects by the probability of being in the post-randomization subgroup and the randomly assigned study therapy. Subjects are weighted to appropriately represent the population by creating a pseudopopulation. These probabilities can be based on factors that change over time. Suppose among those undergoing PCI, those assigned to the treatment arm had higher comorbidities than those assigned to the control arm. Ideally, we would have for a person in the treatment arm with a high comorbidity, a similar person on the control arm with a high comorbidity—as you would imagine in a randomized setting, but due to confounding factors, that counterpart is “missing.” Weights are then estimated based on patient characteristics, which can be incorporated into the analysis in order to create a more representative sample. Their validity relies on an assumption that weights can be well estimated by observed characteristics. A limitation therefore is that only measured clinical features can be included in the weighting, and non-measured factors may be important.

Hirji et al. offer two alternative solutions to what they term outcome-based subgroup analyses—a special case of post-randomization subgroup analyses, where subgroups are defined based on an outcome and the interest is in examining a different outcome [1]. An example they provide is evaluating quality of life among survivors of a cancer trial. The solutions involve single imputation of possible outcomes for those missing data. In the example of the quality of life study, this would involve imputing the worst value for those who did not survive, instead of excluding them from the analysis. Single imputation methods, however, are shown to be biased as they do not appropriately account for the uncertainty of the imputation process [32, 33]. In this case, where the data are likely to be not missing at random, we agree with Hirji and others that standard multiple imputation, which relies on a missing at random assumption, is not recommended [1]. However, multiple imputation-based methods can still be applied under a not-missing-at-random assumption, although its implementation increases in complexity [33]. Although more complicated, we recommend this approach over that proposed by Hirji et al. [1]. In particular, we recommend considering plausible distributions from which to impute, as Hirji et al. did in the single imputation context, but we further recommend averaging over the uncertainty of these choices [1, 33].

Finally, we recommend performing sensitivity analyses, as Pieper and others did, to demonstrate variability in findings as modeling assumptions vary [20]. Consistency across analyses strengthens the evidence, and disparate results should trigger further examination to enable insight into what may be influencing particular findings. They also provide a fuller context for the reader to interpret. Below, we summarize these guidelines.

## Our Guidelines for Pre- and Post-Randomization Subgroup Analyses

Yusuf and others provided excellent and detailed guidelines for analyzing pre-randomization subgroups [5]. This was followed more recently by Wang and others who provided comprehensive guidelines for reporting findings from these subgroup analyses [7]. We build upon these recommendations and expand them to include guidelines for post-randomization subgroup analyses as well (Table 2).

**Table 2:** Guidelines for performing subgroup analyses that build upon those provided by Yusuf et al. (1991)

Design
State plausible subgroup hypotheses and note which are defined by post-randomization features
Rank hypotheses in order of plausibility
Calculate power. Consider adjusting the design if necessary
State methods for analysis and be specific (i.e., include functional form of all relevant variables—continuous or categorical and if categorical, specify the cut-offs)
State conclusions that can be drawn from the analysis plan and any resulting decisions that may occur as a consequence of the findings
Analysis
Use tests of interaction to formally assess heterogeneity of effects
Distinguish between a priori and data-driven hypotheses. Do not present *p* values for data-driven hypotheses
Adjust for multiplicity for a priori subgroup analyses
If post-randomization:
• Consider causal inference tools
• Consider method for incorporating time into model
• Consider sensitivity analyses, where several models are fit and results compared across models
Interpretation and reporting
Report findings corresponding to primary hypothesis
Report the number of a priori hypotheses tested
Report the number of data-driven hypotheses examined
Interpret findings in the context of previous studies and/or similar data from other trials, and based on biological plausibility
Consider pooling findings for subgroup analyses with other studies
Consider external data set where methods can be applied to replicate findings and/or provide code for fitting model, particularly if a post-randomization analysis was conducted so that other investigators can more easily replicate

### Pre-Specify and Define the Subgroups in the Design Phase

Like Yusuf et al., we too recommend starting in the design phase [5]. Here, one should state a priori all biologically plausible subgroup analyses that should be considered and rank the corresponding hypotheses in order of importance. See the recent publication by Thomas and Peterson describing their recommendations on pre-specifying analyses in the observational setting where similar discussions are taking place [34].

### Limit the Number of Subgroups

We recommend limiting this number, keeping in mind that methods to adjust for multiplicity should be applied to ensure meaningful interpretation. Consequently, too many pre-specified hypotheses may make it impossible to observe any interesting differences when adjusting for multiplicity. To aid in this, calculating the power with a conservative Bonferroni correction and meaningful effect size will provide insight into feasibility. If the power is inadequate, the investigator should consider the importance of including the analysis (the ranking will be helpful here). At this point, the investigator can decide whether to adjust the study design accordingly by increasing the sample size to allow for its evaluation. We recommend writing out the specific analysis plan for the subgroup analyses. This will also help with the subsequent guideline of determining precisely what can be concluded from the proposed analysis. Oftentimes, it is not clear exactly what conclusions one can derive from a proposed analysis. Stating this beforehand can clarify whether the choice of subgroup analysis as defined is appropriate and can prevent the generation of a posteriori subgroup analyses, which should be treated differently in the interpretation and given much less weight in importance.

### Formally Test for Heterogeneity Using Statistical Tests of Interaction and Methods to Correct for Multiplicity

Widespread in the literature are inappropriate comparisons across levels of subgroups where investigators incorrectly conclude there are or are no differences in treatment effect by informally comparing *p* values or point estimates. Formal tests of interaction will appropriately address whether effects are heterogeneous beyond variation expected by chance. Use of such methods is encouraged and easy to use with any statistical software. Also, we agree with Yusuf and others that while *p* values and tests of significance can be applied to those subgroup analyses that were pre-specified, they are not meaningful for those that are data-driven, and should therefore not be reported [5]. Instead, data-driven hypotheses should be tested in an external or independent data set. We additionally recommend applying methods to control either the family-wise error rate or the false-discovery rate.

### Explore Causal Inference Methods for Post-Randomization Subgroups

We recommend exploring the use of causal inference methods like instrumental variables, marginal structural models, or propensity scores. We acknowledge, however, that these tools are limited. In particular, if unobserved confounders exist, or an instrument that fulfills necessary criteria is not identified, the problem may be intractable. In addition, it may be feasible to consider approaches to incorporate time into the analysis, as Pieper and others did, for example, by not considering events that occurred prior to the occurrence of PCI [20].

### Perform Sensitivity Analysis for Post-Randomization Subgroups

Due to the high risk of providing biased results, sensitivity analyses that vary models/assumptions should be performed to show how these assumptions may influence the findings. The variation in findings will help the reader to weigh the evidence.

### Report on All Analyses Performed, Whether Positive or Negative

For the interpretation and reporting phase, we borrow ideas from both Yusuf et al. and Wang et al. [5, 7]. Wang and others recommend reporting the number of pre-specified subgroup analyses performed as well as the number of data-driven analyses [7].

### Focus on the Findings that Correspond to the Primary Hypothesis

We agree with Yusuf et al., Wang et al., and Pieper et al. that the emphasis of the report should center around the main findings—those that correspond to the primary hypothesis [5, 7, 20]. All other analyses for which the study was not designed should receive considerably less attention.

### Provide a Context in Which the Reader Can Interpret the Study’s Findings

Yusuf et al. recommend putting in context the study’s findings with those of previous studies of similar design/data and making sense of any discrepancies [5]. This involves reflecting on the study’s design and/or methodological limitations, and/or the appropriateness/generalizability of the study population. Taking this one step further may be to pool the findings with other studies in a meta-analysis or pooling the data with that from other studies in a pooled analysis (if appropriate) to summarize findings across studies [35]. One can also set up next steps for other authors to replicate the findings in either another population to assess generalizability or in another data set of a similar population to validate the findings. Providing code to do this is key as details provided in most papers given their word limitations may not be sufficient for others to apply the exact methods employed. Precise implementation of the methods used is crucial for replication.

## Conclusions

Post-randomization subgroup analyses face the same challenges as those encountered in pre-randomization subgroup analyses. There is one crucial difference. Because post-randomization subgroups are defined by a feature measured at some point during the study, estimates of treatment are potentially biased. Analyses that thoughtfully incorporate time into the model and/or that attempt to minimize the bias should be considered. Issues raised for pre-randomization subgroup analyses should also be considered. And importantly, for all subgroup analyses, the findings from the primary hypothesis should be emphasized whether negative or positive. All other findings should be considered hypothesis-generating. Replication and/or pooling are important tools that can be used to strengthen evidence for positive findings that result from such analyses. Evidence must reach a certain level, however, before it can be considered in influencing clinical practice, and subgroup analyses—post or pre-randomization—do not reach this level. They do have their place, however, and can appropriately affect the direction of new science, if performed thoughtfully. Our study helps to synthesize the key issues to consider in such analyses and provides general guidelines for approaching subgroup analyses.
